# Role of surface composition in morphological evolution of GaAs nano-dots with low-energy ion irradiation

**DOI:** 10.1186/1556-276X-7-552

**Published:** 2012-10-04

**Authors:** Tanuj Kumar, Manish Kumar, Govind Gupta, Ratnesh Kumar Pandey, Shammi Verma, Dinakar Kanjilal

**Affiliations:** 1 Inter-University Accelerator Centre, Aruna Asaf Ali Marg, New Delhi, 110067, India; 2 National Physical Laboratory, Dr K S Krishnan Road, New Delhi, 110012, India; 3 Nanotechnology Application Centre, University of Allahabad, Allahabad, 211002, India

**Keywords:** Ion irradiation, Nano-dots, XPS, AFM

## Abstract

The surface chemistry of GaAs (100) with 50-keV Ar^+^ ion beam irradiation at off-normal incidence has been investigated in order to elucidate the surface nano-structuring mechanism(s). Core level and valence band studies of the surface composition were carried out as a function of fluences, which varied from 1 × 10^17^ to 7 × 10^17^ ions/cm^2^. Core-level spectra of samples analyzed by X-ray photoelectron spectroscopy confirmed the Ga enrichment of the surface resulting in bigger sized nano-dots. Formation of such nano-dots is attributed to be due to the interplay between preferential sputtering and surface diffusion processes. Valence band measurement shows that the shift in the Fermi edge is higher for Ga- rich, bigger sized nano-dots due to the partial oxide formation of Ga. ‘One-dimensional power spectral density’ extracted from atomic force micrographs also confirms the significant role of surface diffusion in observed nano-structuring.

## Background

Well-organized ordered semiconductor nanostructures build the basis for many technological applications as well as for the development of future optoelectronic, electronic, and magnetic devices [1,2]. For the fabrication of ordered semiconductor nanostructures, a number of techniques, i.e., photolithography [3], sublithography [4], scanning probe tip [5], ion beam sputtering [6], and molecular beam epitaxial process (using partial capping of nano-dots) [7,8] have been reported. Among them, low-energy ion irradiation has proven to be a cost-effective, one-step approach for the generation of nanostructures with different topographies at the semiconductor surfaces. No requirement of any kind of masks/templates for nanostructure creation makes this technique even more advantageous over other techniques. By controlling the irradiation parameters, well-ordered nanostructures like one-dimensional ripples, regular arrays of dots and pits, etc. can be evolved in semiconductor materials [6,9-11].

In general, the formation of nano-dots or ripples depends on whether the ion beam is incident on the surface at normal condition or at off-normal irradiation. A lot of experimental [9-11] as well as theoretical [12,13] studies have been performed to understand the basic mechanism(s) of the formation of ripples and/or dots on surfaces subjected to energetic ion irradiation. The most common effect of ion irradiation is the direct transfer of energy and momentum to surface atoms by ion-atom collision, leading to adatom diffusion at the surface. Such studies of low-energy ion irradiation are mainly carried out on Si and Ge materials; however, such effects on compound semiconductors (i.e., GaAs, InP, GaSb, etc.) have been sparsely reported. The fabrication of nano-dots on compound semiconductor surfaces induced by ion irradiation is of particular interest due to the higher possibility of production of well-organized nano-dots under the effect of preferential sputtering [14]. As far as the applications are concerned, the fabrication of semiconductor nano-dots on GaAs surface is of immense importance in the field of optoelectronics, photonics, recording media, and optical applications. We have earlier reported the formation of nano-dots on GaAs surfaces [15]. The preferential sputtering of As atoms as compared to Ga atoms was found to play a crucial role in the formation of nano-dots on GaAs (100) surface. However, the role of size evolution of nano-dots in the context of preferential sputtering and diffusion-induced agglomeration has not been studied. This work is an extension of our previous work to understand the role of surface chemistry on the size evolution of nano-dots. Such kind of study is important to understand the role of different ion irradiation-induced surface modification mechanisms in the case of compound semiconductors.

In this work, the core-level and valence band spectra of Ar^+^-induced self-assembled nano-dots on GaAs (100) surface are presented. The power spectral density has been extracted from atomic force microscopy (AFM) analysis to understand the mechanism involved in surface nano-structuring. Possible mechanisms involved in surface nano-structuring of GaAs (100) are presented to correlate the size evolution and compositional variation of nano-dots.

## Main text

### Experimental

The synthesis procedure of nano-dot formation on GaAs (100) surface has already been reported in our previous article [15]. In brief, 50-keV Ar^+^ ion beam irradiation of GaAs (100) samples were carried out at an angle of 50° with respect to the surface normal inside the vacuum chamber with a pressure of 6.7 × 10^−7^ mbar. During the experiment, the ion beam current density was stabilized at 15 μA/cm^2^. The samples (pristine and irradiated with fluences of 1 × 10^17^, 3 × 10^17^, and 7 × 10^17^ ions/cm^2^) were studied by X-ray photoelectron spectroscopy (XPS) to study the surface chemistry of GaAs (100) as a function of irradiation fluence. The spectra were taken on a PerkinElmer (PHI-1257) XPS system (PerkinElmer Corporation, Eden Prairie, MN, USA) using a Mg anode (source energy = 1,253.6 eV). The deconvolution was performed using the program XPSPEAK4.1 in which we used the Tougaard baseline subtraction. One-dimensional power spectral density (1D-PSD) has been extracted from AFM images at different fluences of ion beam.

## Discussion

**Figure 1:**
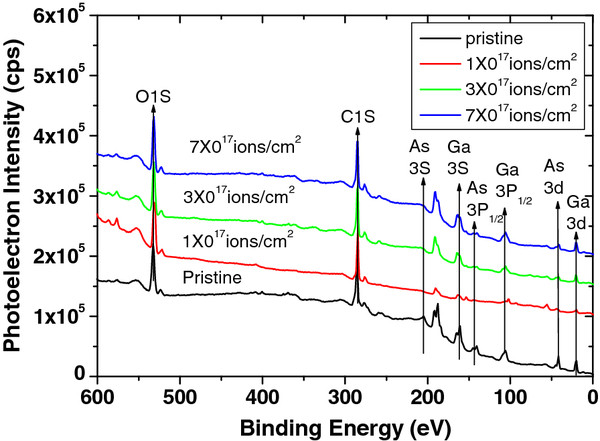
General scan XPS spectra of pristine and irradiated samples at various fluences used.

**Figure 2:**
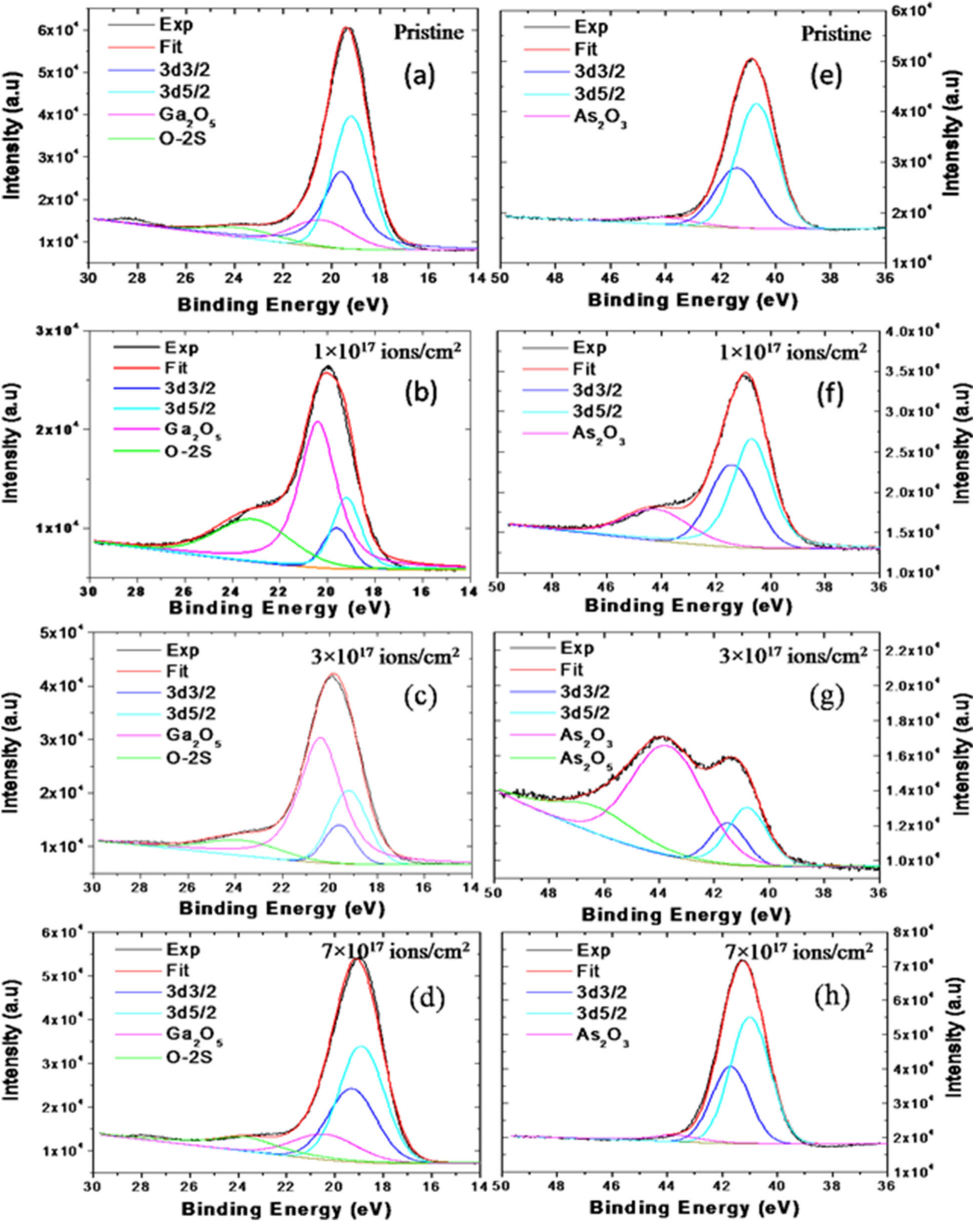
**Core-level XPS spectra of Ga and As elements.** Pristine samples (**a**, **e**) and samples irradiated at the fluences of 1 × 10^17^ (**b**, **f**), 3 × 10^17^ (**c**, **g**), and 7 × 10^17^ ions/cm^2^ (**d**, **h**) (a, b, c, and d correspond to Ga, whereas e, f, g, and h correspond to As).

XPS study was performed to characterize the surface composition of pristine and irradiated GaAs samples. The general scans of pristine and irradiated samples are presented in a binding energy range of 0 to 600 eV in Figure 1. These scans show that the samples are primarily composed of Ga and As elements. The presence of C and O is mainly due to surface contamination. The binding energy curves of Ga 3*d* and As 3*d* core-level spectra were decomposed into different components as shown in Figure 2, each of them showing the different chemical environments of the atoms near the surface. The high-resolution XPS binding energy curves for Ga 3*d* core levels are shown in spectra a to d, and As 3*d* core levels are shown in spectra e to h, where spectra ‘a and e,’ ‘b and f,’ ‘c and g,’ and ‘d and h’ correspond to the pristine sample and irradiated samples with fluences of 1 × 10^17^, 3 × 10^17^, and 7 × 10^17^ ions/cm^2^, respectively. To study the surface composition, the XPS spectra were fitted by a superposition of model components, where the energy separation presents the *d*_3*/*2_-*d*_5*/*2_ spin-orbit splitting. The core-level splitting binding energies for Ga 3*d*_5/2_ and 3*d*_3/2_ are 19.2 and 19.6 eV, respectively, and those for As 3*d*_5/2_ and 3*d*_3/2_, 40.7 and 41.4 eV, respectively. From Figure 2a,b,c,d, one can also see the formation of Ga_2_O_5_ and O2*s* with a peak position at 20.4 and 23.7 eV, respectively. Figure 2a,b,c,d shows that the area as well as the intensity of the Ga_2_O_5_ peak continuously rises up to the fluence of 3 × 10^17^ ions/cm^2^ and then falls down for the fluence of 7 × 10^17^ ions/cm^2^. Figure 2e,f,g,h shows the additional peaks of As_2_O_3_ and As_2_O_5_ at the BE of 44.2 and 45.7 eV, respectively, with core-level As binding energy peaks. The trend of rise in area as well as intensity of the As_2_O_3_ peak is observed up to the fluence of 3 × 10^17^ ions/cm^2^ and falls again for the fluence of 7 × 10^17^ ions/cm^2^.

**Figure 3:**
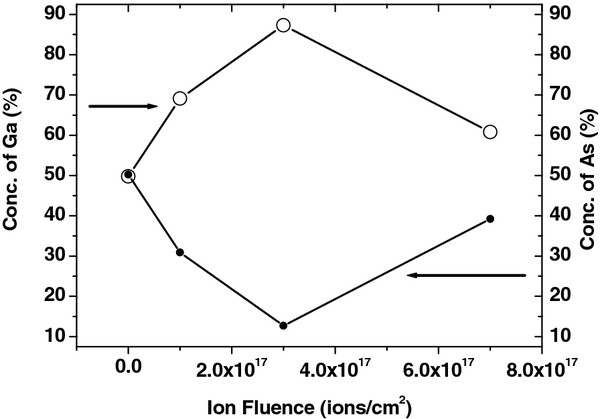
**Variation in chemically bonded Ga/As elements for pristine and irradiated GaAs samples at different fluences.** Empty and filled circles correspond to concentration of Ga and As, respectively, as pointed by arrows in the figure.

From Figure 2, the numerical results on the weights of different spectral components of Ga 3*d* and As 3*d* for pristine and irradiated samples have been calculated, and the ratio of chemically bonded Ga/As as a function of irradiation fluences is presented in Figure 3. It has been found that the surface compositions of Ga and As are 49.8% and 50.2%, respectively, for the unirradiated GaAs. After irradiation at the fluence of 1 × 10^17^ ions/cm^2^, the concentration of Ga rises up to 69.1% as compared to that of As which is 30.9%. For further irradiation at the fluences of 3 × 10^17^and 7 × 10^17^ ions/cm^2^, the observed surface compositions are 87.3% and 60.8% for Ga and 12.7% and 39.2% for As, respectively.

**Figure 4:**
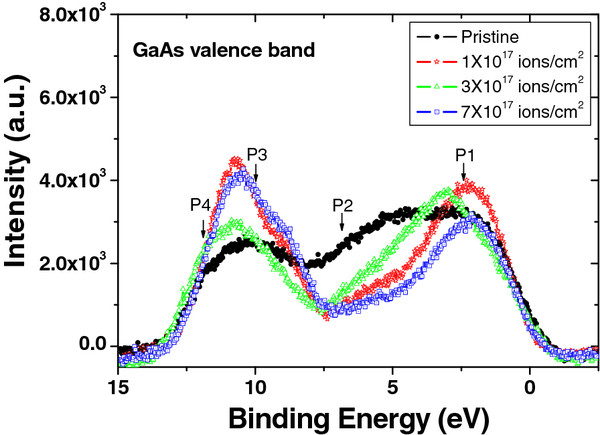
**Valence band spectra of pristine and Ar**^**+**^-**ion irradiated GaAs (100) at different fluences.**

Figure 4 shows the valence band spectra of pristine and irradiated GaAs (100) samples with respect to fluences. Inelastic scattering background corrections have been done in the observed spectra. Our experimental observed valence band spectrum is co-related with the valence band density of states curve proposed by Chelikowsky and Cohen [16]. The shape of the valence band spectrum is appreciably modified with the ion beam irradiation. The most intense peak centered at 2.5 eV (P1) corresponds to *p*-like Ga-As bonding orbital. However, the less intense peaks at 7.0 eV (P2) correspond to the mixed *sp* state. The combination of Ga-O-Ga (P3) and quasi-pure *s*-like As states (P4) are observed at 10.3 and 11.7 eV, respectively. Our results show that only the peaks that correspond to *p*-like Ga-As bonding orbitals are well resolved. From the figure, one can see that the position of peak P1 appreciably shifted towards the higher binding energy side up to 2.8 eV for the fluence of 3 × 10^17^ ions/cm^2^, while this shift is negligible for fluences of 1 × 10^17^and 7 × 10^17^ ions/cm^2^. The shift in peak position attributed the change in density of states. From the core-level XPS results, we have attributed that the preferential sputtering of As results in the Ga-rich surface, which changed into oxides of Ga. This is attributed that the ‘O’ have higher electronegativity as compared to ‘As,’ which make stronger bonds with Ga, and results in the shift in valence band density of states towards higher energy [17]. The shifts in *p*-like Ga-As bonding orbitals (P1) of irradiated samples at different fluences are inconsistent with their surface composition.

**Figure 5:**
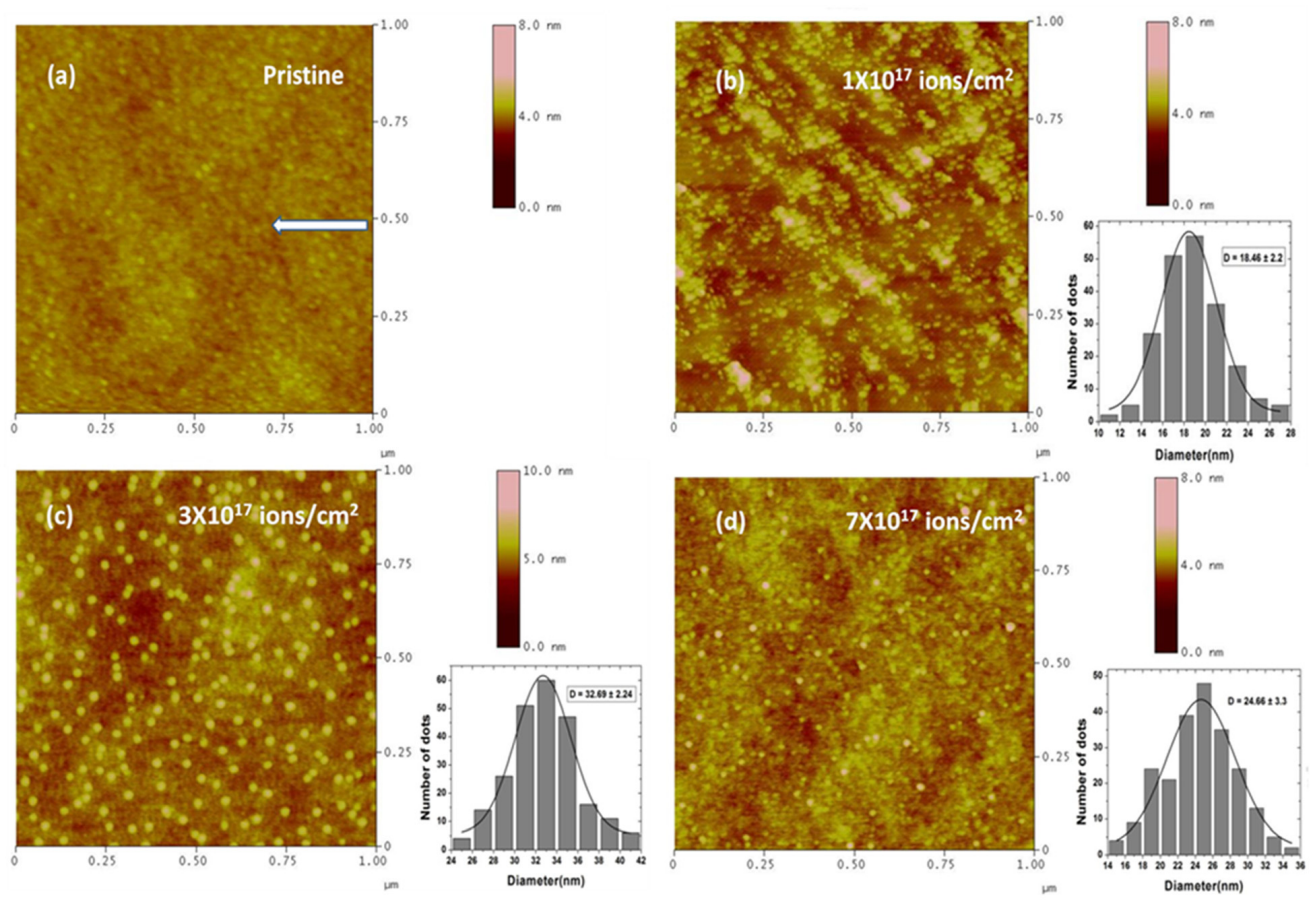
**AFM micrographs.** (**a**) Pristine; 50-keV Ar^+^-irradiated substrates of GaAs (100) at an angle of 50° with respect to surface normal at different fluences: (**b**) 1 × 10^17^, (**c**) 3 × 10^17^, and (**d**) 7 × 10^17^ ions/cm^2^. The arrow in the figure indicates the projection of ion beam direction on the surface. Nano-dot size distribution is shown in the insets.

**Figure 6:**
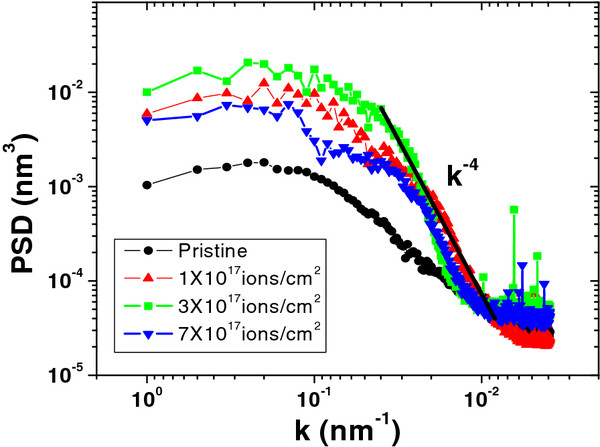
**Log-log plot of 1D-PSD spectral density as a function of spatial frequency.** This was obtained from AFM micrographs for pristine and for irradiated GaAs samples at different fluences.

The morphological evolution of the different nano-dots was studied on GaAs (100) at different ion fluences in our earlier work [15]. In this work, we reported the size enhancement of nano-dots from 18 ± 2 to 32 ± 2 nm when the fluence is increased from 1 × 10^17^ to 3 × 10^17^ ions/cm^2^, and further increase in fluence to 7 × 10^17^ ions/cm^2^ leads to the decrease in average size of nano-dots to 24 ± 3 nm, as shown in Figure 5. To understand the surface nano-structuring mechanism(s) of GaAs with ion fluence, 1D-PSD spectra have been extracted from the AFM images presented in Figure 6. Following the method suggested by Yang et al. [18], the PSD of a surface is calculated using the Fourier transform of height-height correlation function of the AFM data with $G\left(r\right)=<{\left(h_{i}-h_{j}\right)}^{2}>$, where $h_{i}$ and $h_{j}$ are the heights at ith and jth points separated by distance *r* over the surface. The 1D-PSD spectra provide information about the mechanisms which are dominating during surface nano-structuring. Log-log plot of 1D-PSD spectra versus frequency *k* is plotted for pristine and Ar^+^-irradiated GaAs samples at fluences of 1 × 10^17^, 3 × 10^17^, and 7 × 10^17^ ions/cm^2^ as shown in Figure 6. One could easily neglect the corrugation effect by considering the surface as flat for the separation of points by much larger than the correlation length *k*_0_. So, we can divide the power spectra into two distinct regimes of low-frequency part for (*k* < *k*_0_) that is associated with the white noise due to random arrival of ions, and high-frequency regime for (*k* > *k*_0_), which represents the characteristic of the surface morphology according to power-law dependence [19]:

(1) $$\text{PSD}=A{k^{-}}^{\delta }\text{,}$$

where *A* is the proportionality constant and *δ* is a real number. The observed values of *δ* obtained by fitting the power spectra at high frequencies are shown in Figure 6 for all irradiated samples. The observed slope of the PSD tail for irradiated samples reveals *k*^−4^ dependences which is the characteristic behavior of surface diffusion [19]. So, we could conclude that the Ga agglomeration at the surface is strongly dominant by the thermal/ion beam-induced surface diffusion mechanism.

**Figure 7:**
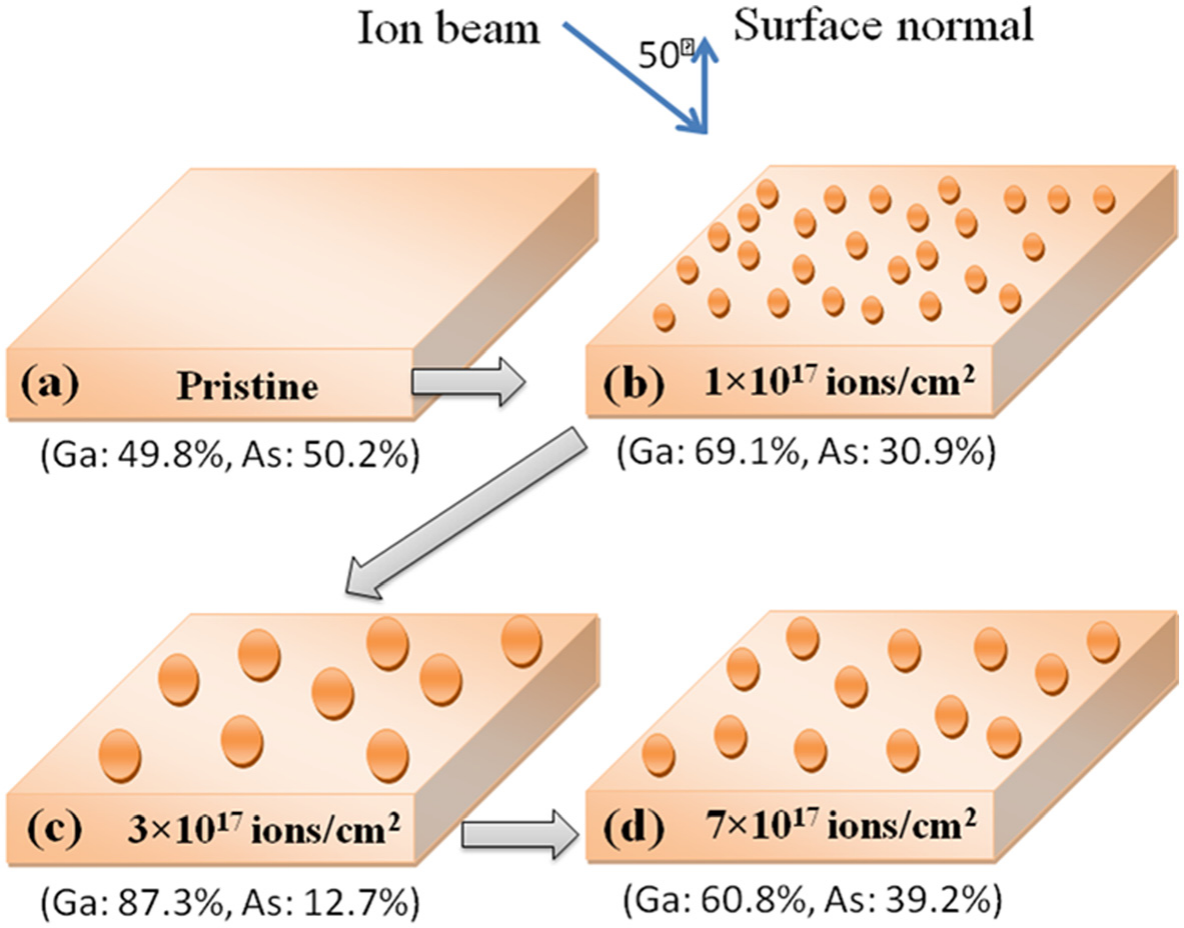
**The schematic view of the surface nano-structuring under off-normal irradiation on GaAs.** Irradiation-induced nano-structuring is governed by competitive processes of preferential sputtering and surface diffusion.

The schematic view of the surface nano-structuring under the used experimental conditions is presented in Figure 7. From the schematic view, we present that the ion irradiation of featureless pristine GaAs (100) samples (Figure 7a) at the fluence of 1 × 10^17^ ions/cm^2^ results in the formation of nano-dots (Figure 7b) under two competitive processes of preferential sputtering of As as compared to Ga and surface diffusion of Ga adatoms. Figure 7c shows that further irradiation at the fluence of 3 × 10^17^ ions/cm^2^ results in bigger sized nano-dots due to higher enrichment of Ga as compared to As. Strain relaxation by the fragmentation of these dots results in the decrease in size with the fall of concentration of Ga at the surface as shown in Figure 7d. Formation of ripples and nano-dots usually arises as a result of the interplay between sputtering-induced roughening and smoothing by surface diffusion [12,20]. The process of surface diffusion occurs by two means, namely ion-induced diffusion and thermal diffusion. For the distinction of these two diffusions, it is very necessary to calculate local temperature at the surface. Nakata has reported that in the case of Si irradiated by low-energy ions, the sample temperature during irradiation depends on the input power density of the ion beam and heat dissipation by radiation or conduction loss [21]. Using the same formulae, we calculated the maximum input power density in the present case to be 0.75 W/cm^2^. Considering the heat dissipation to be mainly due to radiation in vacuum, the maximum wafer temperature (*T*) can be calculated using the relation *P*_b_ = *σε*(*T*^4^ − *T*_s_^4^), where *P*_b_ is the input power density by ion beam, *ε* is the effective emittance of the GaAs wafer of 0.5, *σ* is the Stefan-Boltzmann constant of 5.67 × 10^−12^ W/cm^2^, and *T*_s_ is the surrounding temperature in the chamber. The calculated wafer temperature is 710 K during ion beam irradiation. However, if heat loss by conduction in ladder is considered, the wafer temperature may be reduced by a couple of hundreds of Kelvin resulting in a final temperature similar to that observed by Nakata [21]. These Ga adatoms (melting point of Ga is 300 K) undergo a diffusion process due to thermal diffusion and/or momentum transferred by incident ions leads to the formation of islands or nanoscale dots on the surface. For the present experimental conditions, using the SRIM 2008 [22], the sputtering yields of Ga and As are estimated to be 4.6 and 10 atoms/ion, respectively, which show that the sputtering of As is more preferential as compared to Ga.

In the present experiments, we observed the nonuniform variation of size, roughness, and density of dots as a function of ion fluence as shown in Table 1. This nonlinear behavior in surface roughness and density of dots has been arisen due to coarsening of dots by Ga agglomeration mechanism due to preferential sputtering of As from 1 × 10^17^ to 3 × 10^17^ ions/cm^2^ and fragmentation of nanoscale dots from 3 × 10^17^ to 7 × 10^17^ ions/cm^2^ due to strain relaxation as observed in the Raman spectra (reported earlier in [15]). There are a lot of discrepancies between theories and experiments about the parameters that control the size as well as the density of dots. Wang et al. [23] observed the formation of nano-dots with an average diameter of 22 nm on GaAs surface by Ar^+^ ion sputtering at about 1.2 keV at normal incidence. Datta et al. [24] reported that the average size of nano-dots is of the order of 200 nm on GaAs (100) by 60-keV Ar^+^ ion irradiation at 60° of ion incidence with respect to surface normal. They also observed the decrease in size of dots with increase in ion fluence. Facsko and coworkers [25] observed that as the sputtering continues, the density of dots decreases, whereas the diameter of the dots increases, until a regular organized pattern of dots is formed with the maximum density attainable with an assigned set of experimental parameters. Paramanik et al*.*[26] also identified the irregular behavior of size variation of InP nano-dots with increase in fluence, which is explained by the combination of two processes, *viz.* inverse ripening and fragmentation of nano-dots due to strain production and relaxation by ion irradiation.

**Table 1:** Size, density of dots, and surface RMS roughness values are presented at different fluences

**Fluence (ions/cm**^**2**^**)**	**Size of dots (nm)**	**RMS roughness (nm)**	**Density (dots/cm**^**2**^**)**
Pristine	-	0.2	-
1 × 10^17^	18.4 ± 2.2	0.5	8 × 10^10^
3 × 10^17^	32.6 ± 2.2	0.6	2.8 × 10^10^
7 × 10^17^	24.6 ± 2.2	0.3	5.8 × 10^10^

RMS, root-mean-square.

From the calculated XPS results, we found that the surface composition of Ga and As is in the ratio of around 1:1 (Ga = 49.8% and As = 50.2%) for the unirradiated GaAs. As the sample was irradiated at the fluence of 1 × 10^17^ ions/cm^2^, the features of Ga become more intense as compared to those of As (Ga = 69.1% and As = 30.9%), due to preferential sputtering of As atoms. Thus, the irradiation with Ar^+^ of GaAs at the fluence of 1 × 10^17^ ions/cm^2^ causes surface enrichment with Ga, resulting in the agglomerated Ga-enriched nanoscale dots/islands with a size of 18 nm (Figure 1) because of thermal as well as ion-induced diffusion. When increasing the fluence up to 3 × 10^17^ ions/cm^2^, the surface composition is in the ratio of 87.3% (Ga) and 12.7% (As) which results in the formation of bigger islands/nano-dots with an average size of 30 nm. Here, there can be some contribution of ion irradiation at off-normal condition which can lead to the significant enhancement in the composition ratio of Ga/As. A similar kind of enhancement in compositional ratio by off-normal irradiation was also observed by Pan et al. [27]. Interestingly, for further irradiation at the fluence of 7 × 10^17^ ions/cm^2^, a reverse effect of preferential sputtering of Ga is observed in which the surface concentration of Ga and As is 60.8% and 39.2%, respectively. This fall in concentration of Ga from 87.3% (for fluence of 3 × 10^17^ ions/cm^2^) to 60.8% (for fluence of 7 × 10^17^ ions/cm^2^) at the surface causes the decrease in size of nano-dots (from 30 to 24 nm as observed by AFM analysis) which is in good agreement with AFM results. Gnaser et al. [28] proposed that the elemental composition ratio and steady state of surface composition is strongly a function of ion energy, flux, and fluence. They found that the elemental composition ratio of Ga to As (*C*_Ga_/*C*_As_) is more for 1-keV energy of Ar as compared to 500 eV for the ion beam flux of the order of 10^12^ ions/cm^2^ under the effect of high mobility of atoms. However, in our experiment, the used ion beam energy is 50 keV which is quite high as compared to 1 keV, and the flux is two orders higher (10^14^ ions/cm^2^) which might result in high *C*_Ga_/*C*_As_ for the used fluences. Mohanty et al. [29] also have reported the high surface composition ratio of In to P (In/P = 3.63) for 100-keV Ar^+^ ion beam irradiation of InP. Pan et al. [27] also have observed the In to P surface composition ratio as In/P = 2.2 for ion beam irradiation of 1 to 5 keV Ar^+^.

It was also seen that the surface morphological evolution with sputtering of compound semiconductors was solely affected by the concentration gradient at the surfaces [6]. According to Sigmund's theory of sputtering [30], the lower binding energy of As than Ga and mass difference between Ga (mass = 69.7 amu) and As (mass = 74.9 amu) cause the slow ejection rate of Ga as compared to As by ion irradiation of GaAs. Thus, the preferential sputtering of As causes the Ga enrichment at the surface of GaAs after irradiation. Indeed, the development of In-rich cone-like structures also has been reported earlier [31] by Ar^+^ ion irradiation. Som et al. [32] and Sulania et al. [33] also have reported the surface composition study of the nanostructured InP by ion beam irradiation, but they have not correlated the size variation of nano-dots with the surface composition. Here, we have successively correlated that the surface composition plays a crucial role in the controlled production of size as well as density of dots over the surface by ion beam irradiation.

## Conclusion

In this work, the variation in surface chemistry of GaAs (100) with 50-keV Ar^+^ ion beam irradiation at off-normal incidence has been presented in order to elucidate the surface nano-structuring mechanism(s). XPS study has proven that the change in the irradiation fluences leads to the formation of nano-dots via preferential sputtering of As as compared to Ga and surface diffusion of Ga adatoms due to thermal and/or ion-induced diffusion. The observed size of surface nano-dots after irradiation in the fluence regime of 1 × 10^17^ to 7 × 10^17^ ions/cm^2^ is directly correlated to the Ga enrichment of the surface. Valence band study also confirms that the composition of the surface remains a critical parameter to vary the density of states due to partial oxidization of Ga. Thus, the controlled evolution of nano-dots can be achieved in compound semiconductors by tailoring the surface composition by low-energy ion beam irradiation.

## Competing interests

The authors declare that they have no competing interests.

## Authors’ contributions

TK performed the experiments and wrote the manuscript. MK analyzed the experimental results and helped in writing the manuscript. GG carried out the XPS characterization. SV and RKP helped during the irradiation of samples and in PSD analysis. DK participated and contributed in the design of study and coordination. All authors read and approved the final manuscript.
